# Supplementary material for: Total Fatty Acid and Polar Lipid Species Composition of Human Milk
Source: Nutrients. 2021 Dec 29;14(1):158. doi: 10.3390/nu14010158 (PMC8747362; doi:10.3390/nu14010158)
Supplement: Supplementary file 1 [file nutrients-14-00158-s001.zip › nutrients-1522793-supplementary.pdf]

**Supplemental Table S1.** Characteristics of study population (n=789, HUMIS cohort).

| Characteristic | N | Mean | SD | Min | Max |
|---|---|---|---|---|---|
| Maternal age at pregnancy starts (years) | 789 | 29.6 | 4.7 | 16.0 | 43.0 |
| Maternal height (cm) | 777 | 167.3 | 6.2 | 149.0 | 199.0 |
| Pre-pregnancy BMI (kg/m²) | 761 | 25.0 | 4.8 | 16.4 | 47.9 |
| Gestational weight gain (kg) | 756 | 15.1 | 6.0 | -7.0 | 38.0 |
| Total fatty fish dinners in one-year (servings)[1] | 731 | 26.0 | 28.2 | 0 | 282.3 |

[1]Defined as total fatty fish dinners in past year.

**Supplemental Table S2.** Percentages of the major PC species in all samples and comparison of the percentages between the lowest and highest-fat quartiles.

| %PCs | Total sample (n=664) | Lowest FA quartile (n=166) | Highest FA quartile (n=166) | *p*-value |
|---|---|---|---|---|
| | | presumably containing only saturated FAs | | |
| PCaa.C32:0 (16:0/16:0) | 14.77±3.72 | 15.33±3.82 | 14.47±3.26 | 0.026 |
| PCaa.C34:0(16:0/18:0) | 13.52±4.65 | 14.20±4.70 | 13.02±4.41 | 0.019 |
| PCaa.C36:0 (18:0/18:0) | 0.53±0.20 | 0.56±0.23 | 0.50±0.17 | 0.004 |
| | | presumably containing monounsaturated FAs | | |
| PCaa.C32:1 (16:0/16:1) | 1.29±0.39 | 1.28±0.35 | 1.29±0.29 | 0.723 |
| PCaa.C34:1 (16:0/18:1) | 11.00±2.15 | 10.66±2.19 | 11.38±1.94 | 0.002 |
| PCaa.C36:1 (18:0/18:1) | 6.30±1.15 | 6.41±1.27 | 6.35±1.13 | 0.612 |
| PCaa.C38:1 (20:0/18:1) | 0.59±0.40 | 0.60±0.40 | 0.56±0.37 | 0.315 |
| | | presumably containing polyunsaturated FAs | | |
| PCaa.C34:2 (16:0/18:2) | 6.97±1.96 | 6.46±1.91 | 7.35±1.96 | **$3.90 \times 10^{-4}$*** |
| PCaa.C36:2 (18:0/18:2) | 25.22±5.47 | 24.60±5.40 | 25.88±4.80 | 0.024 |
| PCaa.C38:2 (20:0/18:2) | 2.00±0.82 | 2.07±0.81 | 1.90±0.60 | 0.028 |
| PCaa.C38:3 (20:0/18:3) | 1.75±0.44 | 1.76±0.46 | 1.75±0.37 | 0.806 |
| PCaa.C36:3 (18:0/18:3) | 2.37±0.86 | 2.12±0.80 | 2.60±0.80 | **$1.00 \times 10^{-6}$*** |
| | | presumably containing LCPUFAs | | |
| PCaa.C36:4 (16:0/20:4) | 0.58±0.26 | 0.50±0.23 | 0.64±0.22 | **$2.56 \times 10^{-8}$*** |
| PCaa.C38:4 (18:0/20:4) | 1.38±0.36 | 1.32±0.37 | 1.44±0.34 | 0.002 |

*Significant with a Bonferroni adjusted p-value≤0.001. Data presented as mean±SD. Values are expressed as mol% of total phosphatidylcholine. PCs: phosphatidylcholine species, FA: fatty acid, LCPUFAs: long chain polyunsaturated FAs.

**Supplemental Table S3.** Percentages of the major SM species in all samples and comparison of the percentages between the lowest and highest FA quartiles.

| %SM | Total sample (n=664) | Lowest FA quartile (n=166) | Highest FA quartile (n=166) | *p*-value |
|---|---|---|---|---|
| | | presumably containing saturated FAs | | |
| SM.C32:1(18:1/14:0) | 0.81±0.29 | 0.85±0.29 | 0.79±0.26 | 0.048 |
| SM.C34:1(18:1/16:0) | 7.93±1.50 | 8.40±1.70 | 7.62±1.36 | **$9.00 \times 10^{-6}$*** |
| SM.C35:1(18:1/17:0) | 0.47±0.20 | 0.50±0.13 | 0.44±0.11 | **$2.00 \times 10^{-5}$*** |
| SM.C36:1(18:1/18:0) | 11.19±2.07 | 11.36±2.21 | 11.12±1.93 | 0.294 |
| SM.C37:1(18:1/19:0) | 0.52±0.14 | 0.54±0.14 | 0.50±0.13 | 0.021 |
| SM.C38:1(18:1/20:0) | 5.60±0.77 | 5.60±0.71 | 5.60±0.73 | 0.928 |
| SM.C39:1(18:1/21:0) | 0.86±0.54 | 0.85±0.16 | 0.81±0.16 | 0.013 |
| SM.C40:1(18:1/22:0) | 31.22±5.80 | 30.90±5.76 | 31.65±5.61 | 0.226 |
| SM.C41:1(18:1/23:0) | 3.94±0.58 | 3.91±0.63 | 3.94±0.53 | 0.557 |
| SM.C42:1(18:1/24:0) | 16.39±2.69 | 15.97±2.63 | 16.76±2.77 | 0.008 |
| SM.C43:1(18:1/25:0) | 0.50±0.09 | 0.50±0.09 | 0.50±0.09 | 0.916 |
| | | presumably containing unsaturated FAs | | |
| SM.C36:2(18:1/18:1) | 0.72±0.22 | 0.77±0.24 | 0.69±0.21 | 0.005 |
| SM.C38:2(18:1/20:1) | 0.53±0.20 | 0.52±0.14 | 0.53±0.14 | 0.568 |
| SM.C40:2(18:1/22:1) | 2.00±0.45 | 1.93±0.40 | 2.05±0.46 | 0.008 |
| SM.C41:2(18:1/23:1) | 0.80±0.20 | 0.86±0.21 | 0.76±0.18 | **$7.00 \times 10^{-5}$*** |
| SM.C42:2(18:1/24:1) | 10.70±2.59 | 10.90±2.43 | 10.61±2.39 | 0.348 |
| SM.C43:2(18:1/25:1) | 0.50±0.53 | 0.51±0.13 | 0.50±0.12 | 0.012 |
| SM.C42:3(18:1/24:2) | 1.01±0.62 | 1.03±0.26 | 0.99±0.25 | 0.089 |

*Significant with Bonferroni adjusted p-value≤0.001. Data presented as mean±SD. Values are expressed as mol% of total sphingomyelin (SM)

**Supplemental Table S4.** Regression of LCPUFAs containing %PCs with % FAs in the lowest and highest-FA quartiles.

| Total FA | Dependent Variable (% PCs) | Independent Variable (%FAs) | $R^2$ | B (95%CI) | β | *p*-value |
|---|---|---|---|---|---|---|
| | | | Lowest-FA Quartile(n=166) | | | |
| | %PCaa.C36:4 | | 0.095 | | | |
| | | %C16:0 | | 0.009(0.003 - 0.016) | 0.217 | 0.004 |
| | | %C20:4n-6 | | 0.215(0.058 - 0.372) | 0.202 | 0.008 |
| | %PCaa.C38:4 | | 0.162 | | | |
| | | %C18:0 | | -0.009(-0.024 - 0.007) | -0.079 | 0.277 |
| | | %C20:4n-6 | | 0.694(0.431 - 0.956) | **0.380** | **$5.46 \times 10^{-7}$** |
| | %PCaa.C36:5 | | 0.244 | | | |
| | | %C16:0 | | 0.001(0.000 - 0.002) | 0.182 | 0.008 |
| | | %C20:5n-3 | | 0.071(0.051 – 0.092) | **0.470** | **$1.19 \times 10^{-10}$** |
| | %PCaa.C38:5 | | 0.256 | | | |
| | | %C18:0 | | -0.003(-0.008 – 0.001) | -0.103 | 0.131 |
| | | %C20:5n-3 | | 0.242(0.176 – 0.309) | **0.486** | **$2.67*10^{-11}$** |
| | %PCaa.C38:6 | | 0.226 | | | |
| | | %C16:0 | | 0.004(0.001 – 0.006) | 0.223 | 0.001 |
| | | %C22:6n-3 | | 0.049(0.034 – 0.065) | **0.430** | **$3.72 \times 10^{-9}$** |
| | %PCaa.C40:6 | | 0.180 | | | |
| | | %C18:0 | | -0.002(-0.007 – 0.004) | -0.047 | 0.512 |
| | | %C22:6n-3 | | 0.078(0.051 – 0.104) | **0.417** | **$2.88 \times 10^{-8}$** |
| | | | Highest-FA quartile (n=166) | | | |
| | %PCaa.C36:4 | | 0.100 | | | |
| | | %C16:0 | | 0.007(-0.001 – 0.016) | 0.127 | 0.090 |
| | | %C20:4n-6 | | 0.390(0.200 – 0.581) | **0.302** | **$8.10 \times 10^{-5}$** |
| | %PCaa.C38:4 | | 0.148 | | | |
| | | %C18:0 | | -0.007(-0.024 – 0.010) | -0.061 | 0.406 |
| | | %C20:4n-6 | | 0.745(0.456 – 1.035) | **0.371** | **$1.00 \times 10^{-5}$** |
| | %PCaa.C36:5 | | 0.289 | | | |
| | | %C16:0 | | 0.000(-0.001 – 0.001) | 0.042 | 0.522 |
| | | %C20:5n-3 | | 0.083(0.062 – 0.103) | **0.534** | **$1.48 \times 10^{-13}$** |
| | %PCaa.C38:5 | | 0.431 | | | |
| | | %C18:0 | | -0.002(-0.005 – 0.002) | -0.058 | 0.328 |
| | | %C20:5n-3 | | 0.338(0.277 – 0.399) | **0.649** | **$2.76 \times 10^{-21}$** |
| | %PCaa.C38:6 | | 0.538 | | | |
| | | %C16:0 | | 0.004(0.001 – 0.006) | 0.210 | 0.002 |
| | | %C22:6n-3 | | 0.068(0.051 – 0.086) | **0.503** | **$2.01 \times 10^{-12}$** |
| | %PCaa.C40:6 | | 0.301 | | | |
| | | %C18:0 | | -0.004(-0.010 – 0.001) | -0.105 | 0.114 |
| | | %C22:6n-3 | | 0.127(0.095 – 0.159) | **0.523** | **$3.81 \times 10^{-13}$** |

%PCaa: %diacyl-phosphatidylcholine, %FAs: % fatty acids, $R^2$: coefficient of determination, B(%CI): Unstandardized coefficient with 95%confidence interval, β: standardized regression coefficient.
